# Characterization of the Wheat-*Psathyrostachys huashania* Keng 2Ns/2D Substitution Line H139: A Novel Germplasm With Enhanced Resistance to Wheat Take-All

**DOI:** 10.3389/fpls.2020.00233

**Published:** 2020-03-10

**Authors:** Shengsheng Bai, Fengping Yuan, Hanbing Zhang, Zhenyue Zhang, Jixin Zhao, Qunhui Yang, Jun Wu, Xinhong Chen

**Affiliations:** ^1^Shaanxi Key Laboratory of Plant Genetic Engineering Breeding, College of Agronomy, Northwest A&F University, Yangling, China; ^2^Shaanxi Research Station of Crop Gene Resources and Germplasm Enhancement, Ministry of Agriculture, Yangling, China

**Keywords:** take-all, *Psathyrostachys huashania*, *Triticum aestivum*, novel germplasm, H139

## Abstract

Take-all is a devastating soil-borne disease that affects wheat production. The continuous generation of disease-resistance germplasm is an important aspect of the management of this pathogen. In this study, we characterized the wheat-*Psathyrostachys huashania* Keng (*P. huashania*)-derived progeny H139 that exhibits significantly improved resistance to wheat take-all disease compared with its susceptible parent 7182. Sequential genomic *in situ* hybridization (GISH) and multicolor fluorescence *in situ* hybridization (mc-FISH) analyses revealed that H139 is a stable wheat-*P. huashania* disomic substitution line lacking wheat chromosome 2D. Expressed sequence tag-sequence tagged site (EST-STS) marker and Wheat Axiom 660K Genotyping Array analysis further revealed that H139 was a novel wheat-*P. huashania* 2Ns/2D substitution line. In addition, the H139 line was shown to be cytologically stable with a dwarf phenotype and increased spikelet number. These results indicate that H139, with its enhanced wheat take-all disease resistance and desirable agronomic traits, provides valuable genetic resources for wheat chromosome engineering breeding.

## Introduction

Wheat (*Triticum aestivum L.*) is one of the most important global food crops, contributing nearly a fifth of the total calories consumed by humans (International Wheat Genome Sequencing Consortium (IWGSC), 2018). Wheat production is of great significance to global food security and quality of life. Wheat take-all is a destructive root disease caused by the recently reclassified soil-borne ascomycete fungus *Gauemannomyces tritici* (*G. tritici*), previously referred to as *Gauemannomyces graminis var. tritici* (Walker, 1981; Hernández-Restrepo et al., 2016). This pathogen is widely distributed in wheat-producing areas worldwide (Cook, 2003; Youn-Sig and Weller, 2013). Generally, wheat roots with take-all disease exhibit black spots followed by gradual necrosis of vasculature tissues, resulting in hindered plant growth and development (Youn-Sig and Weller, 2013). In severe infections, take-all disease causes seedling death or, upon extensive invasion of the crown, the death of mature plants, resulting in reduced yield or crop failure (Penrose, 1985). Take-all disease has a considerable impact on the security of wheat production.

The use of disease-resistant varieties remains an economically viable and environmentally friendly measure to control wheat diseases (McMillan et al., 2014; Wu et al., 2017). Penrose (1985) described a laboratory method for the uniform inoculation of wheat seedlings with take-all pathogen in order to evaluate disease resistance in wheat cultivars. Jia et al. (1982) and Scott et al. (1989) confirmed the variation in take-all resistance in wheat cultivars, and described how cultivars with stronger tillering ability and well-developed roots usually exhibit greater disease resistance. Wang et al. (2012) adopted an optimized disease resistance screening method, based on fungal-colonized agar plugs inoculated 2 cm below wheat seeds in combination with a 0–6 disease grading standard, to identify two varieties with moderate resistance against *G. tritici* (G1037) among 69 Chinese wheat cultivars. McMillan et al. (2014) identified seven *T. monococcum* accessions with moderate resistance to take-all disease in naturally inoculated fields; however, the susceptibility of *T. monococcum* to take-all disease in a seedling pot test does not accurately reflect its disease resistance under field conditions.

The utilization of genetic resources from disease-resistant wild relatives had been proven valuable for wheat improvement (Fu et al., 2003; An et al., 2013; Li et al., 2018). An important aspect of take-all disease resistance research is to identify and develop new resistant germplasm. Oat (*Avena sativa* L.) displays near-complete disease resistance to wheat take-all (*G. tritici*), owing to its root system that can produce the antifungal compound avenacin (Papadopoulou et al., 1999; Qi et al., 2004). However, oats are susceptible to *G. graminis var. avenae*, the causal pathogen of oats take-all (Crombie et al., 1986). Rye (*Secale cereale* L.) is considered more resistant to take-all than common wheat, with a disease resistance ranging from moderate to high (Cook, 2003). *Psathyrostachys huashania* Keng (*P. huashania*) (2n = 14, NsNs) is regarded as a wild relative with high resistance to wheat take-all (Wang and Shang, 2000). *P. huashania* is primarily distributed in the Huashan section of the Qinling Mountains, China (Kuo, 1987; Baden, 1991). This species is favored by researchers because of its cold and drought tolerance, early maturity, perennial traits, dwarf stature, and resistance to wheat take-all (*G. tritici*), stripe rust (*Puccinia striiformis* f. sp. *tritici*), and powdery mildew (*Blumeria graminis* f. sp. *tritici*) (Chen et al., 1996; Fu et al., 2003; Du et al., 2013a).

A previous genetically distant hybridization between wheat line 7182 and *P. huashania* was performed by our research team in 1989, from which the haploid line H881 was successfully isolated (Chen et al., 1991). Subsequently, a series of wheat-*P. huashania* addition lines were developed and characterized (Chen et al., 1996; Wu et al., 2007). Initially, Wang and Shang (2000) used a series of wheat-*P. huashania*-derived offspring to screen for resistance to take-all disease at the seedling stage. This revealed that *P. huashania* displayed high disease resistance, whereas the wheat parental line 7182 was susceptible. In the derived offspring, disease resistance of the addition line H1 was similar to that of *P. huashania*, six addition lines and three substitution lines displayed a moderate level of disease resistance, and the remaining lines were susceptible. More recently, synthetic approaches including cytological observations, GISH, EST-STS marker analyses, morphological analysis, and agronomic trait evaluation have led to the successful identification of a series of wheat-*P. huashania* disomic addition lines, including 1Ns (Du et al., 2014d), 2Ns (Du et al., 2014c), 3Ns (Du et al., 2014b), 4Ns (Du et al., 2014a), 5Ns (Du et al., 2013a), 6Ns (Du et al., 2013b), and 7Ns (Du et al., 2013c), and one disomic substitution line, namely 16-6 (Du et al., 2015). However, the use of wheat-*P. huashanica*-derived offspring for enhanced resistance against wheat take-all disease remains underexplored. It is worth mentioning that: both 16-6 and H139 in this study were derived from the distant hybridization of common wheat 7182 × *P. huashania.* The F_1_ generation H881 (2n = 28, ABDNs) was self sterile. To restore the fertility of the hybrid, 7182 was used as the male parent to back-cross with H811 for two times, and then self-crossed for multiple generations. 16-6 and H139 were selected from BC_2_F_6_ and BC_2_F_8_ generations, respectively (Supplementary Figure S1).

With the development of high-throughput sequencing technology, the number of available SNP in common wheat research has been greatly enriched. At present, the high-density Wheat SNP Arrays provides a powerful tool for the genotype of wheat and its wild relatives as well as their introgression lines. Zhou et al. (2018) used the wheat 660K single nucleotide polymorphism (SNP) array to characterize the complete set of wheat-*A. cristatum* addition/substitution lines according to their homoeologous relationships. This wheat 660K SNP array is highly efficient with a wide range of potential applications. The common wheat 7182 is a self-pollinating plant, whereas *P. huashania* is a cross-pollinating plant. Theoretically, in the homozygous wheat genome background, the wheat chromosomes with homologous *P. huashania* chromosomes should have a higher percentage of heterozygous genotypes than wheat chromosomes for addition/substitution lines, and the substituted wheat chromosomes should have more missed genotype markers and ratio than wheat chromosomes.

In this study, we characterized a wheat-*P. huashanica* derived line H139 that displays wheat take-all disease resistance at both the seedlings and mature stages. Molecular cytological and Wheat 660K Genotyping Array were performed to determine the chromosome composition of H139. Moreover, the morphological traits of H139 were evaluated. Our results indicate that the H139 germplasm resource can serve as bridge material for wheat improvement.

## Materials and Methods

### Experimental Materials

In the wheat take-all disease susceptibility seedling pot bioassay, oat (*Avena sativa* cv. Bayou8, resistant to take-all) and hexaploid wheat (*Triticum aestivum* L. cv. Yangmai158, susceptible to take-all) were used as controls to evaluate the disease resistance of line H139, *P. huashania*, and wheat parent 7182.

In the wheat take-all disease susceptibility mature plant test, wild oat (*Avena fatua* L., resistant), rye (cv. Dongmu70, resistant), and wheat Yangmai158 (susceptible) were used as controls. See Table 1 for details of experimental materials used in this study.

**TABLE 1:** List of experimental materials used in various tests.

Methods	H139	7182	*P. HS*	YM158	16-6	BY8	Wild Oat	DM70	CS
Take-all test on seedling	+	+	+	+	−	+	−	−	−
Take-all test on mature plant	+	+	+	+	−	−	+	+	−
Sequential GISH and mc-FISH	+	−	−	−	−	−	−	−	−
EST-STS makers analysis	+	+	+	−	−	−	−	−	+
Wheat 660K SNP array	+	+	−	−	+	−	−	−	−
Morphological characterization	+	+	−	−	+	−	−	−	−

*Both H139 and 16-6 were wheat-*Psathyrostachys huashania* 2Ns/2D substitution lines; 7182 was wheat parent of H139 and 16-6; *P. HS*: *Psathyrostachys huashania* Keng or *P. huashania*; YM158: Yangmai158, a Chinese wheat cultivar that was as susceptible control to take-all; BY8: Bayou8 (*Avena sativa* L.) was as resistant control to take-all in this study; Wild Oat: *Avena fatua* L., was as resistant control to take-all from field of Yangling, China; DM70: Dongmu70 (*Secale cereale* L.), is a forage rye variety; CS; Chinese Spring. +: the experimental material has been applied to the test, −: no applied.*

G1280, an isolate of *G. tritici* with strong pathogenicity (Feng et al., 2013), was used as the infectious material. These plant materials are preserved at the Shaanxi Key Laboratory of Genetic Engineering for Plant Breeding, College of Agronomy, Northwest A&F University, Shaanxi, China.

### Take-All Disease Susceptibility Test

According to the methods of Wang et al. (2012) with some modification. Specific practices were as follows: equal size seeds were disinfected for 5 min with 70% alcohol, washed three times with sterilized water, then sprouted under 24°C. Fourteen-millimeter diameter flower pots were prepared by filling half full with autoclaved vermiculite. Ten 5-mm diameter agar disks containing take-all infectious material were placed evenly on the vermiculite surface (hyphae down), and then covered with a further 2-cm thickness of vermiculite. Ten sprouted seeds were then sown evenly and covered with thin layer of vermiculite. Three replicates were set up per treatment. All plots were placed in a manual climatic cabinet in a randomized design (16-h day length, temperature 15°C, twice weekly watering). After 22 days of growth, the roots were rinsed gently with running water and disease assessment was performed.

For take-all disease assessment in seedlings, we used the disease level standard 0–6 scale as reported by Wang et al. (2012). Grade 0: no symptoms; Grade 1: 1–10% roots infected; Grade 2: 10–50% roots infected; Grade 3: 50–100% roots infected; Grade 4: seedling stem base appears brown or with black surface covering; Grade 5: yellowing of seedling and dwarfing; Grade 6: seedling death. Take-all disease index was calculated for each pot as follows:

$$\mathrm{Index}\,=\frac{0*X_{0}+1*X_{1}+2*X_{2}+3*X_{3}+4*X_{4}+5*X_{5}+6*X_{6}}{\left(X_{0}+X_{1}+X_{2}+X_{3}+X_{4}+X_{5}+X_{6}\right)*6}*100\%$$

where X_1_, X_2_, X_3_, X_4_, X_5_, X_6_ denotes the number of seedlings at Grades 1–6 take-all disease level.

For the take-all disease susceptibility test on mature plants, 30-cm diameter pots were prepared by filling half full with field soil and smoothing the soil surface. Then, 5-cm diameter disk containing take-all infectious material was placed under wheat seedlings, and the seedlings were transplanted to the prepared pots. Three replicates of five seedlings per pot were created, which were watered twice a week until the grain filling stage. Plants were taken out and the soil was gently washed away from roots. Each root was separated and arranged on a white plate. The total number of roots and the number of infected roots were counted for each plant. The proportion of roots infected in each whole-plant root system was estimated and graded into six categories according to McMillan et al. (2014): no symptoms, slight 1 (1–10% roots infected), slight 2 (11–25%), moderate 1 (26–50%), moderate 2 (51–75%), and sever (more than 75%).

### Sequential GISH and mc-FISH

GISH was conducted to detect *P. huashanica* chromatin of H139. Seeds of H139 were germinated at 24°C on moistened filter paper. Actively growing roots were cut, placed into ice-cold water for 24 h, and then stored in Carnoy’s solution (95% ethanol-acetic acid 3:1, v/v). Chromosome preparation of root tip cells of H139 was performed as Han et al. (2006). The genomic DNA of *P. huashanica* was extracted from fresh leaves using the improved CTAB method (Cota-Sánchez et al., 2006). *P. huashanica* DNA probe was labeled with digoxigenin-11-dUTP using the nick translation method (An et al., 2013). The amount of probe DNA was 2.5 ng, and the ratio of probe/blocking DNA was 1:50 for one slide. Chromosomes were counterstained with 4, 6-diamidino-2-phenylindole (DAPI). Mitotic chromosomes number counts and fluorescent signals were viewed and photographed using a microscope (Olympus BX60) with a Photometrics SenSys CCD camera.

To further distinguish the substituted wheat chromosomes in H139, the slide was eluted in 2 × X_1_ SSC for 2 min, and transferred into 70% alcohol for 20 min, transferred into 100% alcohol for 1 h, and dried in air, and then was prepared to complete mc-FISH. The mc-FISH was performed using pAs1 (Rayburn and Gill, 1986) and pSc119.2 (Mcintyre et al., 1990) as probes. The two probes were mixed according to the proportion of 1:1 with chromosome hybridization. Detailed information about the hybridization patterns of pAs1 and pSc119.2 karyotype data were reported by previous studies (Mukai et al., 1993; Schneider et al., 2003; Tang et al., 2014). The counterstain, detection, and visualization of chromosomes were performed as described above.

### EST-STS Marker Analyses

EST-STS marker analyses was conducted to determine the homologous group of *P. huashania* chromosomes contained in H139 to the 7182 counterpart. Genomic DNA from H139, 7182 and *P. huashanica* were isolated as described previously (Cota-Sánchez et al., 2006). To characterize the genomic composition of H139, 75 pairs of EST-STS markers with polymorphisms between *P. huashania* and 7182 were used (Supplementary Table S1). PCR reaction conditions and procedures were performed as the previously published method (Du et al., 2013a). And the PCR products were separated in 8% non-denaturing polyacrylamide gels and then silver-stained and photographed.

### Wheat 660K Genotyping Array Analyses

The Wheat Axiom 660K Genotyping Array has proven to be a powerful tool for identifying homologous group relationships of alien chromosomes in addition or substitution lines (Zhou et al., 2018). Wheat axiom 660K genotyping array assays were performed by Beijing CapitalBio Technology, Co., Ltd.^1^ H139, 16-6 and 7182 were genotyped using the Affymetrix GeneTitan System. SNP genotype calling and clustering was performed with the Polyploidy version in Affymetrix Genotyping ConsoleTM (GTC) software (Wu et al., 2018). The statistics of heterozygous and missed SNP, as well as the screening and distribution of differences SNP between H139 and 16-6, were completed by Office Excel 2010. Compared with the parent 7182, the Ns chromosome that was homologous to wheat chromosome with the high ratio of heterozygous genotypes was considered additional alien chromosomes. And chromosome with the high proportion of missing genotype data was considered replaced wheat chromosomes in the substitution lines.

### Morphological Trait Evaluation

The morphological traits of H139 and 7182 and 16-6 were evaluated from 2016 to 2018. A randomized complete block design was arranged with three replications. At maturity, 30 plants of each line were harvested to evaluate their morphological traits, including plant height, spike length, spikelets per spike, kernels per spikelet, kernels per spike, and 1000-grain weight. The Duncan’s multiple range procedure was used to compare significant differences between H139 and wheat parent 7182 for morphological traits. Comparisons were made using the analysis of variance procedure in SPSS Statistics v22.

## Results

### Evaluation of H139 Take-All Disease Resistance

**FIGURE 1:**
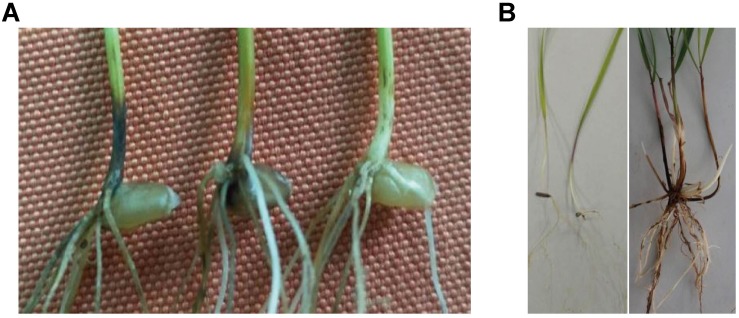
Evaluation of wheat take-all disease susceptibility test. **(A)** Seedlings test of Yangmai 158 (left), 7182 (middle), and H139 (right). **(B)** *P. huashania* performance in the wheat take-all disease susceptibility test performed on seedlings (left) and mature plants (right).

The wheat take-all disease susceptibility test on seedlings was completed in an artificial climate cabinet with controlled temperature, humidity, and light conditions. The disease-resistant control oat (Bayou 8) displayed complete immunity to wheat take-all disease, whereas the susceptible control Yangmai 158 was sufficiently infected with an average disease index (ADI) of 59.4%. The wheat parent 7182 displayed severe disease (ADI 63.0%); however, *P. huashania* showed high take-all disease resistance, with an ADI of only 5.6%, and the wheat-*P. huashania*-derived progeny line H139 was moderately resistant (Figure 1A), with a disease index of 34.9%. The Duncan’s multiple range test showed that the disease resistance of H139 was significantly higher (*P* < 0.01) than that of 7182.

The wheat take-all disease susceptibility test on mature plants was completed in the Northwest Institute of Botany field. The wild oat disease-resistant control displayed the least infection, with an average infected root rate of 9.40% and a disease index of 22.2%, whereas rye (Dongmu 70) displayed moderate infection, with an average infected root rate of 16.5% and a disease index of 42.2%. The susceptible cultivar Yangmai 158 displayed considerable infection, with an average infected root rate of 62.7% and a disease index of 77.3%. The disease index of *P. huashania* was 33.3%, which was between the disease indices of wild oat and rye, and its average infected root rate of 11.3% was close to that of rye (Figure 1B). The wheat parent 7182 exhibited less disease susceptibility than Yangmai158, with an average infected root rate of 44.3% and a disease index of 63.3%. Finally, the disease index of H139 was 43.6% and the average infected root rate was 22.4%, thus indicating that the H139 line has enhanced wheat take-all disease resistance compared with the susceptible parent 7182.

### Sequential GISH and mc-FISH Analyses

**FIGURE 2:**
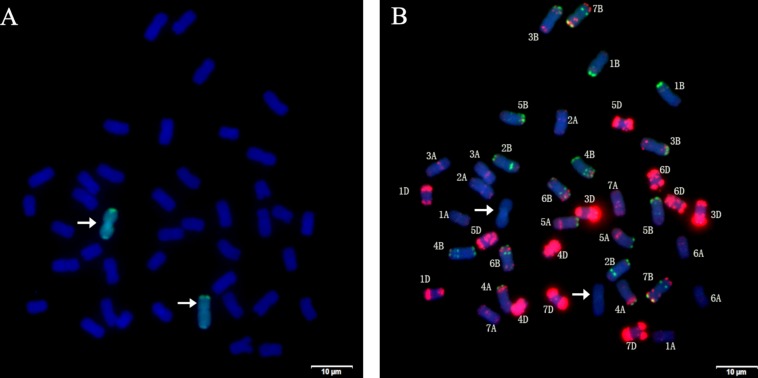
Sequential GISH and mc-FISH analysis of root tip cells of H139. **(A)** GISH analysis of H139 shows two green hybridization signals by using *P. huashania* genomic DNA as probe, and the wheat chromosomes were counterstained with DAPI (blue). **(B)** mc-FISH on the same metaphase after GISH analysis of H139 by using pAs1 (red) and pSc119.2 (green) as probe simultaneously. Arrows indicate the pair of *P. huashania* chromosomes.

Using the genomic DNA of *P. huashania* as probe, GISH analysis of root tip cells of H139 was performed to identify the presence of *P. huashania* chromatin. The result demonstrated that there were 42 chromosomes in H139, of which two showed green fluorescence signals (Figure 2A), thus indicating that H139 contains a pair of *P. huashania* chromosomes. For three consecutive years, H139 was self-pollinated and its offspring were analyzed by GISH, through which no chromosome segregation was observed. Therefore, H139 is considered a genetically stable disomic substitution line.

In order to determine the substituted wheat chromosomes, mc-FISH with probes pAs1 and pSc119.2 was performed in H139. Compared with the karyotype data of pAs1 and pSc119.2, 20 pairs of wheat chromosomes were successfully identified, but the 2D chromosome was not found (Figure 2B). Therefore, we conclude that the wheat 2D chromosome was replaced by a pair of *P. huashania* chromosomes in the H139 line.

### EST-STS Marker Analysis

**FIGURE 3:**
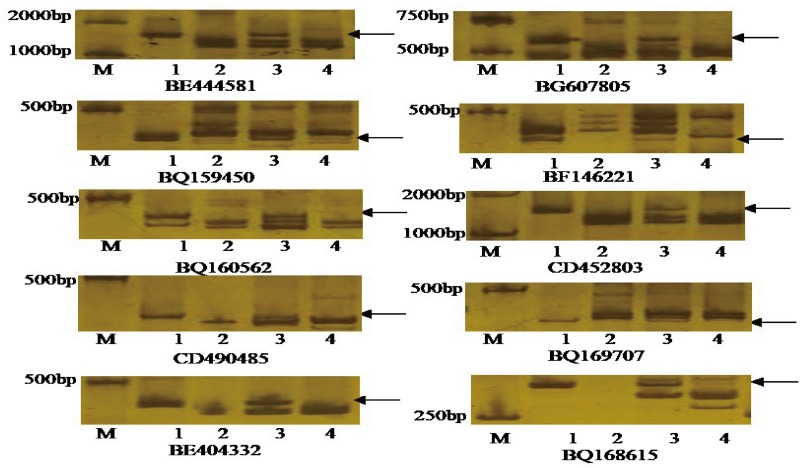
Amplification patterns of the specific EST-STS markers with distributed in wheat homologous group 2. These EST-STS makers amplified the same specific band in H139 and *P. huashania* that was absent in 7182, respectively. M: DNA marker (DL2000); (1) *P. huashania*; (2) 7182; (3) H139; (4) Chinese Spring.

Seventy-five pairs of EST-STS primers, each polymorphic between *P. huashania* and 7182, were used to identify homologous group of *P. huashania* chromosomes in H139. Among these, 10 pairs of EST-STS primers amplified the same specific band in H139 and *P. huashania* that was absent in 7182 (Figure 3). These markers were mainly distributed in the homologous group 2 of common wheat. Therefore, we deduced that the *P. huashania* chromosomes introduced into H139 belonged to homologous group 2. Based on the above results, H139 is considered a wheat-*P. huashania* 2Ns/2D substitution line.

### Wheat 660K SNP Array Analyses

**FIGURE 4:**
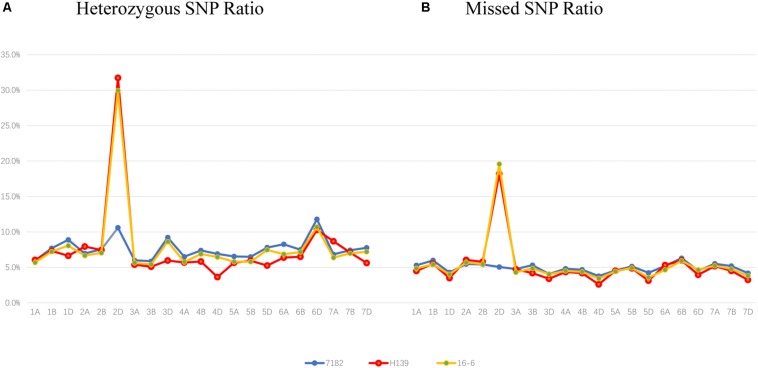
Wheat 660K SNP Array data analysis of 7182 and H139 and wheat-*P. huashania* 2Ns/2D substitution line 16-6. **(A)** The ratio of heterozygous genotypes on each of the wheat chromosomes showed that the ratio of wheat chromosome 2D in H139 and 16-6 was significantly higher than 2D of 7182, and was also the highest ratio compared with other wheat chromosomes. This indicates that *P. huashania* chromosome was 2Ns in H139 and 16-6. **(B)** The missed markers rate of each wheat chromosomes showed that the ratio of wheat chromosome 2D in H139 and 16-6was significantly higher than 2D of 7182, and the missed marker rate of other wheat chromosomes were much lower than that of 2D. This indicates that wheat chromosome 2D was replaced by *P. huashania* chromosome 2Ns in H139 and 16-6.

Compared with EST-STS markers, Wheat Axiom 660K Genotyping Array has a greater number of markers that are distributed more evenly across wheat chromosomes. To further clarify the chromosome composition of H139, the Wheat Axiom 660K Genotyping Array was used for genotyping. The ratio of heterozygous genotypes on each of the wheat chromosomes was determined in H139 and 7182. The results showed that the ratio of heterozygous genotypes of H139 on wheat chromosome 2D was significantly higher than 2D of 7182, and was also the highest ratio (31.73) compared with other wheat chromosomes in H139 and 7182 (Figure 4A). The ratio of heterozygous genotypes of other wheat chromosomes ranged from 3.6 to 10.3%, which was much lower than that of 2D. Therefore, we can confirm that the *P. huashania* chromosome in H139 was 2Ns. This result further validates the results of EST-STS marker analysis.

Moreover, the ratio of missed markers on each chromosome was also calculated in H139 and 7182. The data showed that the missed marker rate of H139 on wheat chromosome 2D was significantly higher than 2D of 7182, and the missed marker rate of other wheat chromosomes ranged from 2.6 to 6.29%, which was much lower than that of 2D (Figure 4B). Therefore, it can be concluded that wheat chromosome 2D is replaced by *P. huashania* chromosome 2Ns in H139, which is consistent with the results of GISH and mc-FISH, and further support the notion that H139 is a wheat-*P. huashania* 2Ns/2D substitution line.

In addition, 16-6, a wheat-*P. huashania* 2Ns/2D substitution line previously reported by Du et al. (2015), had also been re-validated as wheat-*P. huashania* 2Ns/2D substitution line based on the wheat 660K Genotyping Array data (Figure 4). However, 16-6 and H139 were further analyzed and showed that there were 53 4564 (91.5%) same SNP, and 49 909 (8.5%) differential SNP between H139 and 16-6. These differences were widely distributed on 21 pairs of chromosomes (Supplementary Table S2).

**FIGURE 5:**
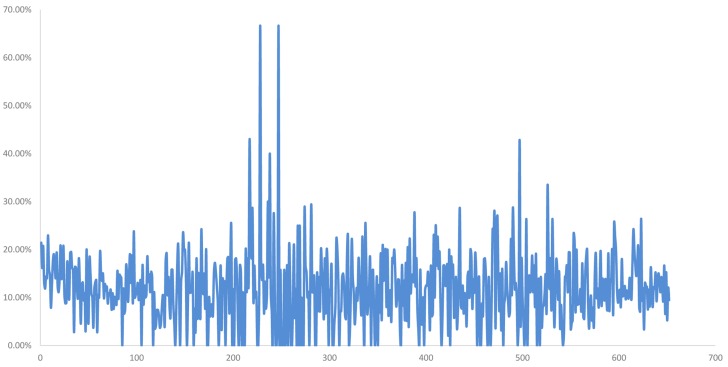
The distribution ratio of differential SNP per Mb on 2Ns(2D) chromosome between in H139 and 16-6. Abscissa is the physical position of SNP on chromosome, and the unit is Mb. The ordinate is the ratio of the number of differential SNP to the total SNP in 1Mb.

Among these, 2785 SNP were distributed on the 2Ns(2D) chromosome, and the distribution ratio of these SNP in 2Ns(2D) was relatively high compared with other chromosomes (Supplementary Table S2). Both H139 and 16-6 are wheat-*P. huashania* 2Ns/2D substitution lines, and their second homologous groups include 2A, 2B, and 2Ns chromosomes without 2D. Therefore, the 2785 SNP likely originated from the 2Ns chromosome. According to the physical position of these SNP on 2D chromosome, the distribution proportion of these differential SNP within 1 Mb was calculated on, the results showed that there was a peak between 200 and 300 Mb (Figure 5). These results demonstrate that there are partial differences in genome between them. And the 2Ns chromosome in H139 is partial differences than 16-6.

### Morphological Trait Evaluation

**FIGURE 6:**
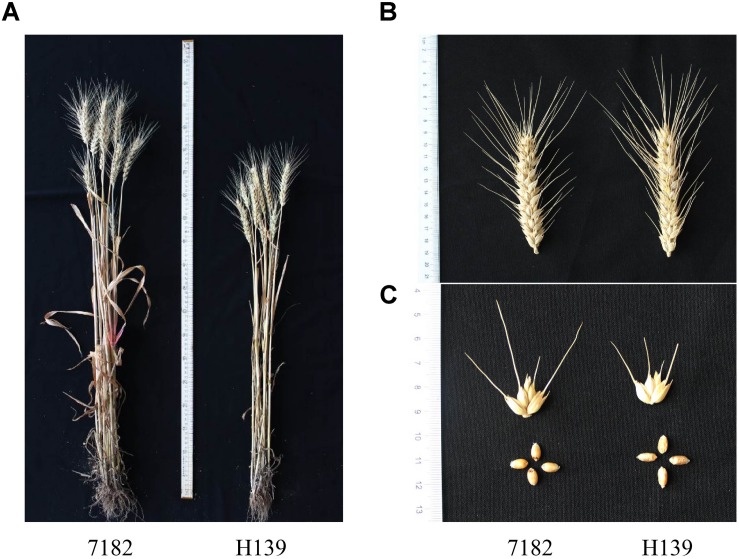
Plant **(A)**, spike **(B)**, spikelet and grain **(C)** morphology of H139 and the parent 7182.

Morphological trait surveys of three consecutive years showed that H139 performed almost identically to wheat parent 7182 regarding seedling growth habit, heading time, spike type, and spike color. Compared with its wheat parent 7182, however, H139 was shorter in plant stature and greater spikelets per spike (Figure 6). Although H139 also exhibited greater spike length, kenels per spike, and 1000-kernel weight than 7182, these differences were not statistically significant (Table 2). Compared with 16-6, there were significant differences between H139 and 16-6 regarding plant height, spike length, spikelets per spike, kernels per spikelet, kernels per spike, and 1000-kernel weight (Table 2).

**TABLE 2:** Data analysis of agronomic traits of 7182 and H139 and 16-6.

	7182		H139		16-6	
Characters	Mean	Range	Mean	Range	Mean	Range
Plant height(cm)	75.3	(65–82)	60**	(51–71)	75.9	(71–80)
Spike length(cm)	8.8	(6.5–10.5)	8.9	(6–11)	12.2**	(9.5–14)
Spikelets per spike	19.9	(16–24)	22.1*	(17–26)	25.9**	(24–27)
Kernels per spikelet	3	(2–4)	3	(2–4)	4	(2–5)
Kenels per spike	55.8	(32–85)	56	(42–84)	75.7**	(66–85)
Thousand-grain weight(g)	46.2	(45.6–47.2)	47.1	(46.7–47.5)	50.5**	(48–53.3)

There were significant differences in means at the **P < 0.01 level and *P < 0.05 level, based on Duncan’s multiple range tests.

## Discussion

Take-all is a typical soil-borne disease more pervasive with continuous cropping that seriously threatens wheat yield and grain quality. Limited germplasm resistant to take-all disease has been identified in common wheat (McMillan et al., 2014). Chromosome engineering is important for crop breeders to develop new disease-resistant germplasm and broaden the genetic base of wheat (Jiang et al., 1993; Zhuang et al., 2015; An et al., 2019). Hollins et al. (1986) found that the triticale was moderate resistance to take-all compared with wheat and rye. And the application of triticale is considered to be an immediately available method to improve wheat take-all resistance. But the introduction of individual pairs of rye chromosomes into wheat genetic background did not significantly slight take-all susceptible. Wang et al. (2003) reported 9 lines with relatively high resistance to take-all in seedling from 19 lines [wheat-*P. huashania* intermedium derivative lines and wheat-*Haynaldia vilosa* L. Schur (2n = 14, VV) intermedium derivatives lines], and then in further field test they found H922-9-12, V2 and V9129-1 expressed high resistance in wheat anthesis period, and H922-9-12 and V2 were high resistance in harvest period. And V2 was a wheat-*Haynaldia vilosa* substitution line, but there was no chromosome location information of take-all disease resistance. *P. huashania* was shown to be a precious germplasm resource with high resistance to take-all disease (Wang and Shang, 2000; Fu et al., 2003). Since the 1980s, our research team has been committed to introducing individual desirable characteristics of *P. huashania* into common wheat by chromosome engineering (Chen et al., 1991). Wang and Shang (2000) reported that *P. huashania* was high resistant to wheat take-all disease and represents a new disease-resistant germplasm resource. Their study reported that seven addition lines, three substitution lines, and two translocation lines with moderate resistance to *G. tritici* through a seedlings susceptibility test. However, there was also no chromosome location information about take-all disease resistance. And the take-all disease resistance of *P. huashanica* and its derivatives in mature plants remains poorly understood.

In this study, we demonstrated through take-all disease resistance seedling assays the disease immunity of control oat, the high-level disease resistance of parent *P. huashanica*, the disease susceptibility of both control Yangmai158 and wheat parent 7182, and the enhanced disease-resistance of line H139. Furthermore, the disease resistance of *P. huashanica* and H139 was analyzed in mature plants, for which we used equal pathogen inoculation. Interestingly, wild oat as a disease-resistant control was infected by *G. tritici*, but the infection was slight. Our results slightly differ from those of McMillan et al. (2014), whereby oat plants were immune to wheat take-all disease in field tests. This discrepancy may be due to differences in experimental oat varieties or inoculation methods. The method used by McMillan et al. (2014) involved natural field pathogen inoculation; however, we used a manual inoculation method that may have resulted in more uniform, high-level pathogen exposure than that occurs in natural field conditions. This greater pathogen inoculation may have resulted in a relatively higher take-all disease index, which is supported by the higher disease indices observed for the susceptible control Yangmai 158 and disease-resistant control rye. Although take-all disease was more prevalent in field trials, H139 maintained a moderate disease index and a lower average disease root rate than the wheat parent 7182. This indicates that H139 plants are resistant to take-all disease at the seedling and mature stage.

Furthermore, H139, which represents a new wheat-*P. huashania* 2Ns/2D substitution line resistant to take-all disease, was characterized by GISH, mc-FISH, molecular marker assays, Wheat 660K Genotyping Array analysis, and morphological traits evaluation. In an earlier study by Du et al. (2014c), the wheat-*P. huashanica* 2Ns disomic addition line 3-6-4-1 was shown to be generally resistant to stripe rust in both seedlings and mature plants. Following this, Du et al. (2015) described the wheat-*P. huashania* 2Ns/2D substitution line 16-6, which exhibited resistance to mixed races of stripe rust (CYR31, CYR32, and SY11-14) in mature plants. The disease resistance observed in these studies was attributed to the *P. huashania* parent, thus demonstrating that the introduction of *P. huashania* chromosome 2Ns could enhance the disease resistance of common wheat. Interestingly, in the pre-experiment of take-all identification, the disease index of 16-6 was greater than 50%, which was considered as a susceptible line. In addition, some morphological characters of H139 and 16-6 are also significantly different. To clarify the reason of their differences, we analyzed the distribution of differential SNP between 16-6 and H139, and found that there could be an obvious peak in 200–300 Mb region between the 2Ns chromosomes of the H139 and 16-6. The difference of SNP in the specific chromosome region of 2Ns may be the cause of the difference of resistance and morphology traits between the two substitution lines. And the uneven distribution of the polymorphic SNP may indicate a deletion or a unexplored chromosome variation on the 2Ns chromosome in one of the substitution lines. The wheat genetic background of the substitution lines 16-6 and H139 are the same as that of 7182 (Du et al., 2015), which provide an opportunity to develop a genetic mapping population for the 2Ns chromosome using the two 2Ns(2D) substitution lines as crossing partners. Producing knock-out mutants by EMS mutagenesis and the application of MutChromSeq approach together with the results of genetic mapping could be a promising way to identify resistance gene against take-all disease. Another important application of line H139 is to hybridize it with other intermediate materials such as hexaploid *triticale* (AABBRR), or directly with rye (Dongmu 70) that resistant to wheat take-all, to develop genetic offspring of multiple genera, and aggregate multiple resistance genes, so as to further improve the resistance to wheat take-all. Meanwhile, hybridization among different genera will further broaden the genetic variation of wheat and enrich the diversity of wheat germplasm materials.

## Conclusion

Through genetically distant hybridization and chromosome engineering, the desirable disease resistance characteristics of *P. huashanica* were introduced into the genetic background of common wheat, which significantly improved take-all disease resistance. Wheat-*Psathyrostachys huashania* Keng 2Ns/2D substitution line H139, as a new germplasm with resistance to wheat take-all and desirable agronomic traits, can be employed as valuable genetic resources for wheat chromosome engineering breeding.

## Data Availability Statement

The wheat 660k array data of H139 has been successfully uploaded to GEO, The accession number is GSE143188. We may view the GSE143188 study at: https://www.ncbi.nlm.nih.gov/geo/query/acc.cgi?acc=GSE143188.

## Author Contributions

SS and XC designed and directed the study. SS, QY, JZ, and JW selected the wheat-*P. huashanica* 2Ns/2D substitution line H139. SS, FY, HZ, and ZZ performed the experiments. SS, HZ, and JW analyzed the data. JW and XC contributed reagents, materials, and analysis tools. SS wrote the manuscript.

## Conflict of Interest

The authors declare that the research was conducted in the absence of any commercial or financial relationships that could be construed as a potential conflict of interest.

## Footnotes

1 http://www.capitalbiotech.com

## Supplementary Material

FIGURE S1 The development process of wheat-*Psathyrostachys huashania* 2Ns/2D substitution line H139 and 16-6.

TABLE S1 75 EST-STS markers with polymorphisms between *P. huashania* and 7182 were used in this study.

TABLE S2 The distribution proportion of differential SNP on chromosomes between H139 and 16-6.

## References

[B1] An D. G. Ma P. T. Zheng Q. Fu S. L. Li L. H. Han F. P. (2019). Development and molecular cytogenetic identification of a new wheat-rye 4R chromosome disomic addition line with resistances to powdery mildew, stripe rust and sharp eyespot. *Theor. Appl. Genet.* 132 257–272. 10.1007/s00122-018-3214-3 30374527

[B2] An D. G. Zheng Q. Zhou Y. L. Ma P. T. Lv Z. L. Li L. H. (2013). Molecular cytogenetic characterization of a new wheat–rye 4R chromosome translocation line resistant to powdery mildew. *Chromosome Res.* 21 419–432. 10.1007/s10577-013-9366-8 23836161

[B3] Baden C. (1991). A taxonomic revision of *Psathyrostachys* (Poaceae). *Nord. J. Bot.* 11 3–26. 10.1111/j.1756-1051.1991.tb01790.x 12152343

[B4] Chen S. Y. Hou W. S. Zhang A. J. Fu J. Yang Q. H. (1996). Breeding and cytogenetic study of Triticum aestivum-*Psathyrostachys huashanica* alien addition lines. *Acta Genet. Sin.* 23 447–452.

[B5] Chen S. Y. Zhang A. J. Fu J. (1991). The hybridization between Triticum aestivum and *Psathyrotachys huashanica*. *Acta Genet Sin.* 18 508–512.

[B6] Cook R. J. (2003). Take-all of wheat. *Physiol. Mo. Plant Pathol.* 62 73–86. 10.1016/s0885-5765(03)00042-0

[B7] Cota-Sánchez J. H. Remarchuk K. Ubayasena K. (2006). Ready-touse DNA extracted with a CTAB method adapted for herbarium specimens and mucilaginous plant tissue. *Plant Mol. Biol. Rep.* 24 161–167. 10.1007/bf02914055

[B8] Crombie W. M. L. Crombie L. Green J. B. Lucas J. A. (1986). Pathogenicity of ‘take-all’ fungus to oats: its relationship to the concentration and detoxification of the four avenacins. *Phytochemistry.* 25 2075–2083. 10.1016/0031-9422(86)80069-3

[B9] Du W. L. Wang J. Lu M. Sun S. G. Chen X. H. Zhao J. X. (2013a). Molecular cytogenetic identification of a wheat-*Psathyrostachys huashanica* Keng 5Ns disomic addition line with stripe rust resistance. *Mol. Breed* 31 879–888. 10.1007/s11032-013-9841-0

[B12] Du W. L. Wang J. Lu M. Sun S. G. Chen X. H. Zhao J. X. (2014a). Characterization of a whea-*Psathyrostachys huashanica* Keng 4Ns disomic addition line for enhanced tiller numbers and stripe rust resistance. *Planta* 239 97–105. 10.1007/s00425-013-1957-2 24085532

[B10] Du W. L. Wang J. Pang Y. H. Li Y. L. Chen X. H. Zhao J. X. (2013b). Isolation and characterization of a *Psathyrostachys huashanica* Keng 6Ns chromosome addition in common wheat. *PLoS One* 8:e53921. 10.1371/journal.pone.0053921 23326537 PMC3542264

[B13] Du W. L. Wang J. Pang Y. H. Wang L. M. Wu J. Zhao J. X. (2014b). Isolation and characterization of a wheat-*Psathyrostachys huashanica* Keng 3Ns disomic addition line with resistance to stripe rust. *Genome* 57 37–44. 10.1139/gen-2013-0199 24564214

[B14] Du W. L. Wang J. Pang Y. H. Wu J. Zhao J. X. Liu S. H. (2014c). Development and application of PCR markers specific to the 1Ns chromosome of *Psathyrostachys huashanica* Keng with leaf rust resistance. *Euphytica* 200 207–220. 10.1007/s10681-014-1145-x

[B15] Du W. L. Wang J. Wang L. M. Wu J. Zhao J. X. Liu S. H. (2014d). Molecular characterization of a wheat-*Psathyrostachys huashanica* Keng 2Ns disomic addition line with resistance to stripe rust. *Mol. Genet. Genomics* 289 735–743. 10.1007/s00438-014-0844-2 24700077

[B11] Du W. L. Wang J. Wang L. M. Zhang J. Chen X. H. Zhao J. X. (2013c). Development and characterization of a *Psathyrostachys huashanica* Keng 7Ns chromosome addition line with leaf rust resistance. *PLoS One* 8:e70879. 10.1371/journal.pone.0070879 23976963 PMC3747159

[B16] Du W. L. Zhao J. X. Wang J. Wang L. M. Wu J. Yang Q. H. (2015). Cytogenetic and molecular marker-based characterization of a wheat-*Psathyrostachys huashanica* Keng 2Ns(2D) substitution line. *Plant Mol. Biol. Rep.* 33 414–423. 10.1007/s11105-014-0761-x

[B17] Feng Y. X. Li W. Sun H. Y. Deng Y. Y. Yu H. S. Chen H. G. (2013). Genetic diversity of Gaeumannomyces graminis var. tritici populations in Huang-Huai winter wheat production region of China. ACTA. Phytophylacica. *SINICA* 40 495–501.

[B18] Fu J. Wang M. N. Zhao J. X. Chen S. Y. Hou W. S. Yang Q. H. (2003). Studies on cytogenetics and utilization of wheat-Psathyrostachys huashanica medium material H8911 with resistance to wheat take-all fungus. *Acta Bot. Boreal. Occident. Sin.* 23 2157–2162.

[B19] Han F. P. Lamb J. C. Birchler J. A. (2006). High frequency of centromere inactivation resulting in stable dicentric chromosomes of maize. *Proc. Nat. Acad. Sci. U.S.A* 103 3238–3243. 10.1073/pnas.0509650103 16492777 PMC1413895

[B20] Hernández-Restrepo M. Groenewald J. Z. Elliott M. L. Canning G. Mcmillan V. E. Crous P. W. (2016). Take-all or nothing. *Stud. Mycol.* 10.1016/j.simyco.2016.06.002 PMC4969266 27504028

[B21] Hollins T. W. Scott P. R. Gregory R. S. (1986). The relative resistance of wheat, rye and triticale to take-all caused by *Gaeumannomyces graminis*. *Plant Pathol.* 35 93–100. 10.1111/j.1365-3059.1986.tb01986.x

[B22] International Wheat Genome Sequencing Consortium (IWGSC) (2018). Shifting the limits in wheat research and breeding using a fully annotated reference genome. *Science* 361:6403. 10.1126/science.aar7191 30115783

[B23] Jia T. Y. Wu Q. B. Ye X. C. Zang F. C. Zhuo Y. N. Gong B. Y. (1982). A preliminary study on the take-all of wheat. *Sci. Agric. Sin.* 15 65–75.

[B24] Jiang J. Friebe B. Gill B. S. (1993). Recent advances in alien gene transfer in wheat. *Euphytica* 73 199–212. 10.1038/hdy.2012.116 23321705 PMC3630809

[B25] Kuo P. C. (1987). *Flora Reipublicae Popularis Sinicae.* Beijing: Science Press, 51–104.

[B26] Li F. Li Y. H. Cao L. R. Liu P. Y. Geng M. M. Zhang Q. (2018). Simultaneous transfer of leaf rust and powdery mildew resistance genes from hexaploid triticale cultivar sorento into bread wheat. *Front. Plant Sci.* 9:85. 10.3389/fpls.2018.00085 29459877 PMC5807375

[B27] Mcintyre C. L. Pereira S. Moran L. B. Appels R. (1990). New Secale cereale (Rye) DNA derivatives for the detection of rye chromosome segments in wheat. *Genome* 33 635–640. 10.1139/g90-094 2262137

[B28] McMillan V. E. Gutteridge R. J. Hammond-Kosack K. E. (2014). Identifying variation in resistance to the take-all fungus, Gaeumannomyces graminisvar.tritici, between different ancestral and modern wheat species. *BMC Plant Biol.* 14:212. 10.1186/s12870-014-0212-8 25084989 PMC4236718

[B29] Mukai Y. Nakahara Y. Yamamoto M. (1993). Simultaneous discrimination of the three genomes in hexaploid wheat by multicolor fuorescence in situ hybridization using total genomic and highly repeated DNA probes. *Genome* 36 489–494. 10.1139/g93-067 18470003

[B30] Papadopoulou K. Melton R. E. Leggett M. Osbourn M. J. D. E. (1999). Compromised disease resistance in saponin-deficient plants. *Proc. Natl. Acad. Sci. U.S.A.* 96 12923–12928. 10.1073/pnas.96.22.12923 10536024 PMC23166

[B31] Penrose L. (1985). Evidence for resistance in wheat cultivars grown in sand culture to the take-all pathogen, Gaeumannomyces graminis var. tritici. *Ann. Appl. Biol.* 107 105–108. 10.1111/j.1744-7348.1985.tb01552.x

[B32] Qi X. Bakht S. Leggett M. Maxwell C. Melton R. Osbourn A. (2004). A gene cluster for secondary metabolism in oat: implications for the evolution of metabolic diversity in plants. *Proc. Natl. Acad. Sci. U.S.A.* 101 8233–8238. 10.1073/pnas.0401301101 15148404 PMC419586

[B33] Rayburn A. L. Gill B. S. (1986). Isolation of a D-genome specific repeated DNA sequence from *Aegilops squarrosa*. *Plant Mo. Biol. Rep.* 4 102–109. 10.1007/bf02732107

[B34] Schneider A. Linc G. Molnár-Láng M. (2003). Fluorescence in situ hybridization polymorphism using two repetitive DNA clones in different cultivars of wheat. *Plant Breed* 122 396–400. 10.1046/j.1439-0523.2003.00891.x

[B35] Scott P. R. Hollins T. H. Summers R. W. (1989). Breeding for resistance to two soil-borne diseases of cereals. *Vortraege Fuer Pflanzenzuechtung* 16 217–230.

[B36] Tang Z. X. Yang Z. J. Fu S. L. (2014). Oligonucleotides replacing the roles of repetitive sequences pAs1, pSc119.2, *pTa-*535, pTa71, CCS1, and pAWRC.1 for FISH analysis. *J. Appl. Genet.* 55 313–318. 10.1007/s13353-014-0215-z 24782110

[B37] Walker J. (1981). “Taxonomy of take-all fungi and related genera and species,” in *Biology and control of take-all*, eds Asher M. J. C. Shipton P. J. (London: Academic Press), 15–74.

[B38] Wang D. B. Wang M. N. Jing J. X. Shang H. S. Li F. X. (2003). Study of resistance in distant hybridigation descendant of wheat to the take-all. *Acta Bot. Boreal.Occident. Sin.* 23 1617–1620.

[B39] Wang M. N. Shang H. S. (2000). Evalution of resistance in *Psathyrostachys huashaica* to wheat take-all fungus. *Acta Uninv. Agric. Boreali Occidentalis* 28 69–71.

[B40] Wang N. Feng Y. X. Du W. Z. Wang Y. Chen H. G. (2012). Virulence of wheat Take-all pathogen and disease resistance of different wheat cultivars. *J. Plant Genet. Resour.* 13 478–483.

[B41] Wu J. Zhao J. X. Chen X. H. Liu S. H. Yang Q. H. Liu W. X. (2007). Cytology characteristic and GISH analysis on the progenies derived from common wheat (*T. aestivum* L.) × *Psathyrostachys huashanica*. *J. Triticeae Crops* 27 772–775.

[B42] Wu J. H. Wang Q. L. Liu S. J. Huang S. Mu J. M. Zeng Q. D. (2017). Saturation mapping of a major effect QTL for stripe rust resistance on wheat chromosome 2B in cultivar Napo 63 using SNP genotyping arrays. *Front. Plant Sci.* 8:653. 10.3389/fpls.2017.00653 28491075 PMC5405077

[B43] Wu J. H. Zeng Q. D. Wang Q. L. Liu S. J. Yu S. Z. Mu J. M. (2018). SNP-based pool genotyping and haplotype analysis accelerate fine-mapping of the wheat genomic region containing stripe rust resistance gene *Yr26*. *Theor. Appl. Genet.* 131 1–16. 10.1007/s00122-018-3092-8 29666883

[B44] Youn-Sig K. Weller D. M. (2013). Take-all of wheat and natural disease suppression: a review. *Plant Pathol. J.* 29 125–135. 10.5423/ppj.si.07.2012.0112 25288939 PMC4174779

[B45] Zhou S. H. Zhang J. P. Che Y. H. Liu W. H. Lu Y. Q. Yang X. M. (2018). Construction of Agropyron, Gaertn. genetic linkage maps using a wheat 660k SNP array reveals a homoeologous relationship with the wheat genome. *Plant Biotechnol. J.* 16 818–827. 10.1111/pbi.12831 28921769 PMC5814592

[B46] Zhuang L. F. Liu P. Liu Z. Q. Chen T. T. Wu N. Sun L. (2015). Multiple structural aberrations and physical mapping of rye chromosome 2R introgressed into wheat. *Mol. Breed.* 35 133.

