# Supplementary figures and images for: Characterization of the Wheat-Psathyrostachys huashania Keng 2Ns/2D Substitution Line H139: A Novel Germplasm With Enhanced Resistance to Wheat Take-All
Source: Front Plant Sci. 2020 Mar 10;11:233. doi: 10.3389/fpls.2020.00233 (PMC7077511; doi:10.3389/fpls.2020.00233)

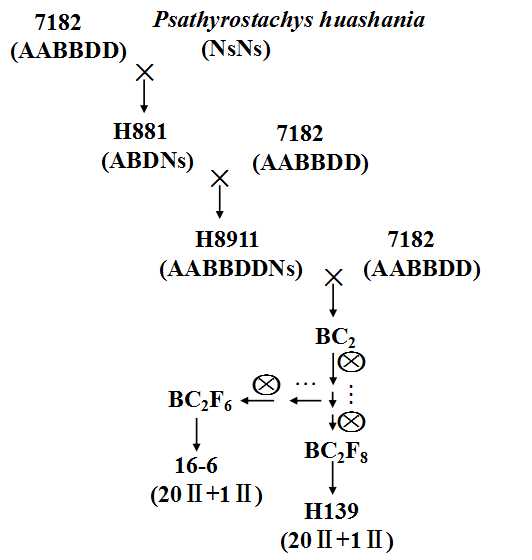

Supplement: FIGURE S1 — The development process of wheat-Psathyrostachys huashania 2Ns/2D substitution line H139 and 16-6. [file Image_1.jpg]
